# SOS Teeth: Age and Sex Differences in the Prevalence of First Priority Teeth among a National Representative Sample of Young and Middle-Aged Adults

**DOI:** 10.3390/ijerph17134847

**Published:** 2020-07-06

**Authors:** Galit Almoznino, Itzhak Abramovitz, Ortal Kessler Baruch, Ron Kedem, Noam E. Protter, Jonathan Levine, Tarif Bader, Nirit Yavnai, Dorit Zur, Eitan Mijiritsky, Boaz Shay

**Affiliations:** 1 Head, Big Biomedical Data Research Laboratory, Hadassah School of Dental Medicine, Hebrew University, Jerusalem 91120, Israel; 2 Department of Oral Medicine, Sedation & Maxillofacial Imaging, Hadassah School of Dental Medicine, Hebrew University, Jerusalem 91120, Israel; boaz@endo.co.il; 3 Department of Endodontics, Hadassah School of Dental Medicine, Hebrew University, Jerusalem 91120, Israel; itzhakab@hadassah.org.il (I.A.); ortalbaruch1@gmail.com (O.K.B.); 4 Medical Information Department, General Surgeon Headquarter, Medical Corps, Israel Defense Forces, Tel–Hashomer 02149, Israel; ron.kedem56@gmail.com (R.K.); Dorit48@mail.idf.il (D.Z.); 5 Chief Dental Surgeon & Head of Forensic Unit, Medical Corps, Israel Defense Forces, Tel–Hashomer 02149, Israel; noamprotter@gmail.com; 6 Department of Prosthodontics, Oral and Maxillofacial Center, Medical Corps, Israel Defense Forces, Tel–Hashomer 02149, Israel; jonathan.levine3@gmail.com; 7 Brigadier General, Surgeon General’s Headquarters, Israel Defense Forces, Ramat Gan 5262000, Israel; drtarifb@gmail.com; 8 Department of Military Medicine, Hebrew University, Jerusalem 91120, Israel; nirityavnai@gmail.com; 9 Medical Research & Academy Section, Medical Corps, Israel Defense Forces, Jerusalem 91120, Israel; 10 Department of Otolaryngology, Head and Neck and Maxillofacial Surgery, Tel–Aviv Sourasky Medical Center, Sackler Faculty of Medicine, Tel Aviv 6139001, Israel; mijiritsky@bezeqint.net; 11 The Maurice and Gabriela Goldschleger, School of Dental Medicine, Tel–Aviv University, Tel Aviv 6139001, Israel

**Keywords:** caries, decayed teeth, electronic medical record, electronic dental record

## Abstract

Background: “SOS teeth” are defined as the first priority teeth for treatment, that have distinct cavitation reaching the pulp chamber or only root fragments are present. Objectives: To assess the prevalence and distribution of SOS teeth with regard to age and sex difference among young to middle-aged adults. Methods: This is a cross-sectional records-based study of a nationally representative sample, consisting of young to middle-aged military personnel, who attended the military dental clinics of the Israel Defense Forces (IDF) for one year. SOS teeth definition corresponds to code number 6 of the “Caries Assessment Spectrum and Treatment (CAST)” as an instrument to assess dental caries. Data pertaining to age and sex were drawn from the central demographic database and that of SOS teeth were obtained from the Dental Patient Record (DPR). Results: The study included 132,529 dental records. The prevalence of patients with SOS teeth was 9.18 % (12,146/132,323). The number of teeth that were found to be SOS teeth was 18,300, i.e., 1.5 SOS teeth per “diseased” patient (18,300/12,146). The mean number of SOS teeth per the whole study population was 0.14 ± 0.52 and the range was 0–20. The mean number of SOS teeth per patient had a statistically significant negative correlation with age (*p* < 0.001; Odds Ratio (OR) = 0.997; 95% confidence interval: 0.997–0.998) and with male sex compared to females (*p* < 0.001; OR = 1.029 confidence interval: 1.023–1.036). Conclusion: Assessment of first priority SOS teeth may be part of the dentist’s work-up. It provides dentists and health authorities with useful information regarding urgent dental care needs to plan dental services.

## 1. Introduction

Dental caries is a diet-dependent, transmissible microbiologically mediated disease that follows an infectious and chronic disease model [1]. According to the Global Burden of Disease (GBD) 2010 study, untreated caries of the permanent dentition was the most prevalent condition worldwide, with a global prevalence of 35% for all ages combined [2]. Owing to its predominance, dental caries is considered the most important oral disease and is of medical, social and economic importance [3].

The most commonly used epidemiological index for assessing dental caries prevalence [4] is the Decayed Missing Filled Surfaces/Teeth (DMFS/T) index by the World Health Organization (WHO) [5]. According to the DMF, which was developed by Klein and Palmer in 1938, a tooth with a carious lesion extending to the dentin is considered a diseased tooth [6]. However, the DMFS/T has several limitations in assessing dental caries. Firstly, it is done without X-ray imaging, and therefore it underestimates real caries prevalence and treatment needs for “Hidden caries” which are visible on a radiograph, but not on visual examination [7,8]. Moreover, some recommend exclusion of teeth with a past caries experience (restored/sealed/missing) from the calculation, since the inclusion of diseased and healthy teeth in one unit of expression may be misleading [9]. 

Another criticism has been made widely for not registering the initial, premorbidity stage [10] as well as advanced stages of dental caries. While the DMF instrument determines whether a cavitated dentin carious lesion is present or not, it does not cover all clinical stages of dental decay [11,12,13]. Given the global epidemic of untreated caries, there was an urgent need to develop scoring systems that will both assess and quantify various stages of caries [14]. To that end, several scoring methods were developed to assess caries activity and severity for use in clinical research, clinical practice and for epidemiological purposes such as the following instruments: the PUFA [14] and its modification: the Pulpal Involvement–Roots–Sepsis Index [15,16], the Nyvad Criteria for Caries Lesion Activity and Severity Assessment [17], the International Caries Detection and Assessment System (ICDAS) [18] and its International Caries Classification and Management System (ICCMS) [12] (ICDAS/ICCMS), the Significant Caries Index (SiC) [19,20] and the Caries Assessment Spectrum and Treatment (CAST) instrument [21] and the CAST severity score [22]. While each of these caries assessment tools have limitations and advantages, they provide insights on disease stage [9].

Of particular importance, the DMF index fails to provide information on the clinical consequences of untreated dental caries, such as pulpal involvement and dental abscess, which may be more serious than the caries lesions themselves [14]. Without treatment, the chance that a person with pulp-involved and abscessed teeth experiences pain or necrosis is high [10]. While the WHO instrument serves as an important screening tool [9], it is crucial to differentiate caries lesions in dentin that can be treated restoratively from those that require more complicated treatment [23]. Assessment of the prevalence of advanced carious lesions provides useful information about the extent of the disease and the urgent dental care required to plan services and compare the impact of treatment over time. For a successful clinical practice, it is essential to prioritize treatment needs that can address the urgent/critical conditions prior to attending less critical dental issues.

Prioritizing treatment needs is particularly necessary for public dental health care systems that include multiple clinics, such as military dental clinics, which must ensure the qualifications of military personnel and improve their quality of life as well as decrease the health related costs for the army. To that end, in the dental branch of the Israel Defense Forces (IDF), following the treatment plan, the dentist determines the first priority teeth for treatment, known as SOS teeth. SOS teeth are defined as teeth that have distinct cavitation reaching the pulp chamber or only root fragments are present. The definition of SOS teeth corresponds to code number 6 of the Caries Assessment Spectrum and Treatment (CAST) instrument to assess dental caries [21]. These are teeth with severe morbidity, that may require pulp capping, root canal treatment or extraction, and therefore should be treated first. 

The present study aimed to assess the prevalence as well as distribution by age and sex of SOS teeth among a unique nationally representative sample of young to middle-aged adults of military personnel in the IDF. To the best of our knowledge, this is the first records-based large scale data study to assess SOS teeth in young- and middle-aged adults in Israel. 

## 2. Materials and Methods 

### 2.1. Study Population

This is part of the Dental, Oral, Medical Epidemiological (DOME), which is a cross-sectional records-based study of a large database. The DOME study consists of the socio-demographic, dental and medical and records of a nationally representative sample, consisting of young to middle-aged military personnel serving in mandatory and career service, who attended all the military dental clinics of the Israel Defense Forces (IDF), between January 1st, 2015 and January 1st, 2016.

### 2.2. Ethical Approval

The study is in line with the STROBE guidelines and was approved by the Medical Corps Institutional Review Board (IRB), approval number: IDF-1281-2013. Due to the retrospective study design, involving only dental records analysis, the IRB approved an exemption from a written informed consent.

### 2.3. Inclusion Criteria

1. Military personnel in mandatory and career service aged 18–50.

2. Existence of records in the central demographic database in the Dental Patient Record (DPR) regarding the subject.

### 2.4. Exclusion Criteria

Lack of records regarding the subject in the DPR and/or central demographic database. 

### 2.5. Data Collection

Data mining was performed by the Medical Information Department, General Surgeon Headquarter, Medical Corps, Tel-Hashomer, Israel. The database is completely anonymous and includes demographic and dental data as follows:

### 2.6. Central Demographic Database

Age and sex were drawn from the IDF’s central demographic database, that records the personal socio-demographic details of the military population [24]. Age and sex were drawn at the mid-study period, i.e., 1 June 2015.

### 2.7. Dental Database

Data on SOS teeth were drawn from the Dental Patient Record (DPR), an electronic dental record (EDR), that stores the complete dental records regarding all dental care in the IDF [25]. The DPR includes the patient’s dental, periodontal and oral records as well as treatment plan and actual treatment done, imaging results and consultation requests. All dental attendees received free and unconditional treatment services since IDF military personnel do not incur any dental expenses [26,27]. Commanders must enable their subordinates full access to dental services, independently of their rank and/or position [26]. Moreover, a baseline evaluation of the dental status is mandatory for combats during their first four months of military training [25].

### 2.8. Dental and Radiographic Examination 

Based on a clinical and radiographic assessment, a treatment plan was made by military dentists and recorded in the DPR. According to the instructions of the dental branch of the IDF Medical Corps, dental examinations were carried out in military dental clinics, in an indoor setting, under conditions such as optimal light, with a dental mirror and ball-ended dental probes and the use of compressed air. The guidelines require routine radiographic assessment included bilateral bitewings for all examinees and supplementary periapical radiographs contingent upon deep caries or former endodontic treatment [25]. Continuous quality assurance assessments and seasonal audits were conducted by the regional military chief dental surgeons to ensure quality assurance of the treatment plan and adherence to the instructions [25].

### 2.9. The Dependent Variable: SOS Teeth

According to these IDF instructions, and following the treatment plan, the dentist determined the first priority teeth for treatment, i.e., the SOS teeth, and records this in the DPR. The definition of SOS teeth is as described in the introduction: distinct cavitation reaching the pulp chamber or only root fragments are present, which corresponds to code number 6 of the CAST instrument to assess dental caries [21]. However, radiographic assessment and the use of compressed air were included in the assessment of SOS teeth, unlike the original CAST assessment which is performed visually by the naked eye, and the use of compressed air is not required [21,28]. 

### 2.10. Statistical Analysis

Data were tabulated and statistical analyses were performed using SPSS software version 22.0 (IBM, Chicago, IL, USA). Numerical variables are presented as means and standard deviations, categorical variables are presented as frequencies and percentages. 

Significance tests between SOS teeth and the independent variables included: Analysis of variance (ANOVA) and general linear model (GLM). Two-tailed statistical significance (α) was considered as *p* < 0.01 due to the large sample size. 

## 3. Results

The study population included 132,323 patients who attended IDF dental clinics during the study period. Table 1 presents descriptive statistics of SOS teeth. The number of patients with SOS teeth among the study population was 12,146. Therefore, of all the patients who were examined (132,323), the prevalence of patients with SOS teeth was 9.18 % (12,146/132,323).

The number of teeth that were found to be SOS teeth was 18,300. Therefore, the mean number of SOS teeth per a diseased patient (i.e., a patient with at least one SOS tooth) was 1.5 (18,300/12,146). In other words, every diseased patient had 1.5 teeth in an SOS condition. The mean number of SOS teeth in the whole study population was 0.14 ± 0.52 and the range was 0–20.

### 3.1. The Distribution of the Number of SOS Teeth per Patient among the Study Population 

Table 2 presents the distribution of the number of SOS teeth per patient in the study population. Most of the study population did not have an SOS tooth (120,177 patients, 90.82%). Of those patients who had SOS teeth (12,146 patients, 9.18 %): most had one tooth in an SOS condition (8323 patients, 6.3%), 2438 (1.8%) had two SOS teeth, 289 (0.6%) had three SOS teeth, 328 (0.2%) and 141 (0.1%) of the patients had 4 and 5 teeth in an SOS condition, respectively. Eighty-six participants (0.06%) among the study population had more than six teeth in an SOS condition. There was only one patient that was found to have 20 teeth in an SOS condition. Of those affected with SOS teeth, 68.52% (8323/12,146) had one tooth in an SOS condition.

### 3.2. The Distribution of SOS Teeth by Age among the Study Population 

**Figure 1:**
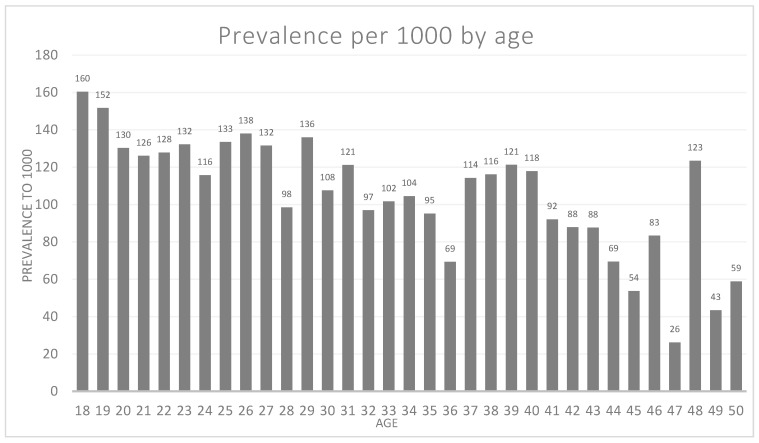
Number of SOS teeth per 1000 by age among the study population.

Table 3 presents the mean number of SOS teeth by age and the rate of SOS teeth per 1000 by age among the study population. The highest prevalence of SOS teeth was at age 18 years (rate 160 per 1000) while the lowest prevalence was found at the age of 47 (rate 26 per 1000). As depicted in Figure 1, the rate of SOS teeth per 1000 by age, there is a decline in the prevalence of SOS teeth with age. 

The parameter of the mean number of SOS teeth per patient had a statistically significant negative correlation with age (General Linear Model: *p* < 0.001; Odds Ratio (OR) = 0.997; 95% confidence interval: 0.997–0.998).

### 3.3. The Distribution of SOS Teeth by Age among Males and Females in the Study Population 

**Figure 2:**
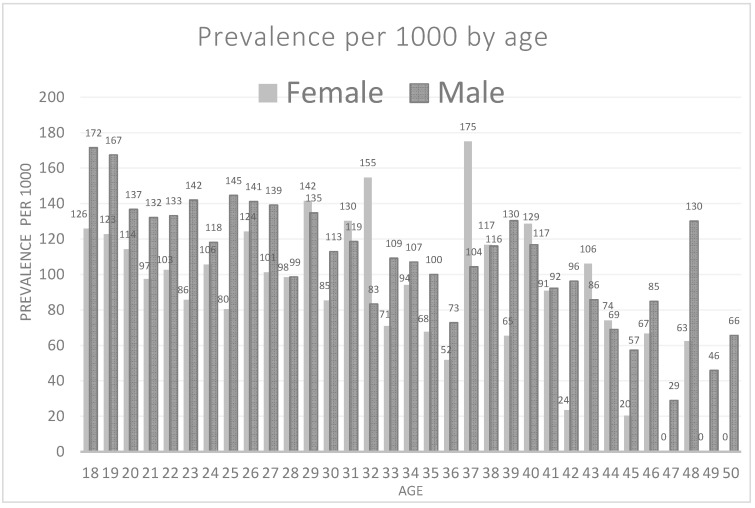
Rate of SOS teeth per 1000 by age among males and females.

Table 4 and Figure 2 present the mean number of SOS teeth and the rate of SOS teeth per 1000 by age and sex. The highest prevalence of SOS teeth for males was at the age of 18 (172:1000) and for females at the age of 37 (175:1000). The range of SOS teeth among women was 0–7, while among males it was 0–20. 

ANOVA analysis demonstrated that SOS teeth had a statistically significant positive association with male sex (*p* < 0.001). The general linear model revealed that the Odds Ratio (OR) and 95% confidence interval for males to have SOS teeth compared to females was 1.029 (1.023–1.036).

## 4. Discussion

SOS teeth represent teeth that have a deep clinical and radiographic tooth decay that may require pulp capping, root canal treatment or extraction and are defined as the teeth to be treated first. This study included a large nationally representative sample that consists of dental and demographic records of 132,323 young to middle-age patients in the IDF. The goals of the present study were to assess the prevalence as well as distribution by age and sex of SOS teeth among those seeking dental care in the IDF. The prevalence of SOS teeth in the study population was 9.18 %. SOS teeth were positively associated with younger age and male sex.

The concept related to SOS teeth can be compared to the concept of the Significant Caries Index (Sic Index), which was proposed to bring attention to the individuals with the highest caries scores in each population under investigation. The Sic Index is calculated as the mean DMFT score of one-third of the population with the highest DMFT caries scores [29]. However, the Sic Index can be calculated only retrospectively, i.e., data about the population is needed to be collected first, while SOS teeth can be applied during the examination to determine the first priority treatment need and allow the dental health authorities to assess urgent dental treatment needs in real-time. Prioritizing the sequence of dental treatment is essential to prevent disease deterioration. While the Sic can be applied in the level of the population, SOS teeth can be counted on an individual as well as in the population level. Moreover, the Sic Index includes previous treatments as counted in the missing and fillings components of the DMFT, while the concept of SOS teeth refers to current dental caries needs.

### 4.1. Prevalence of SOS Teeth

In the present study, composed of young to middle-aged adults, 9.18% of the study population were already in need of endodontic treatment or extraction. Pulp involvement is usually accompanied by episodes of pain, which negatively affect the quality of life of the individual [30] and render the tooth more vulnerable to mortality [22]. Therefore, it is highly significant to understand the consequences of the presence of SOS teeth and deliver immediate dental care.

There is a limited body of research data available describing the SOS teeth to compare our results with other studies. Since code 6 of the CAST corresponds to SOS teeth, we compared our data with the published literature. The prevalence of teeth scored by CAST codes with code 6 varies according to the studied population. de Souza et al. reported that among a sample of 177 children aged 2–6 years and their mothers aged 19–30 years, the most prevalent CAST code in the permanent dentition of the mothers was code 6 (33.3%) [31]. On the other hand, among children aged 6–11 years, 1.2% had one tooth or more and none of the children had three teeth or more with CAST code 6 [23]. Our studied population included both men and women aged 18–50 and was not limited to molars, which may attribute to the difference in the prevalence compared to other studies.

There were only 87 patients who had more than five affected teeth, and only one patient had 20 SOS teeth, which is extremely rare (1/132,323 = 0.00075%) (see Table 2). The advantage of this large sample size is it included the entire patients flow to the dental clinics, and therefore reflects the true distribution of SOS teeth, including the extreme cases in the population, which would otherwise be neglected. These extreme cases represent severe dental morbidity which require special attention from the dentist in terms of risk factors analysis and urgent dental care required.

### 4.2. Associations of Age and Sex with SOS Teeth

Age. In this study, it was found that there is a statistically significant negative correlation between SOS teeth and age, i.e., the number of SOS teeth declines with increasing age. The highest number of SOS teeth was found at the age of 18 years. The OR for age (OR = 0.997) is calculated per year, and although this reflects a relatively weak impact of age on the prevalence of SOS teeth, this impact is not negligible and accumulates over time.

In line with our findings, Doneria et al. observed that 14.28% of primary molars were with CAST code 6, while only 0.6% of permanent molars suffered such morbidity [32]. Moreover, in a cross-sectional study of 4642 patients from the institute of antral Gujarat, it was found that the prevalence of dental caries was the highest (44.3%) in the age group 21–40, while the lowest prevalence was found in the geriatric population above the age of 80 [33]. Diseased teeth in older persons are generally extracted and this can attribute to the declining trend in the disease prevalence with increasing age. In other epidemiologic surveys, it was found that newly erupted teeth are more susceptible to caries, particularly at pit and fissure sites. As children reach early adolescence, there is some indication that the caries incidence slows down. The elderly are mainly at a greater risk for root caries [34] which is the major cause of missing teeth in older adults [35]. In a systematic review, it was found that the burden of untreated caries is shifting from children to adults, with 3 peaks in prevalence at ages 6, 25 and 70 y [36]. 

Sex. In this study, significantly more SOS teeth were found in males compared to females. The OR of sex was 1.029 and although this impact of sex is relatively weak, it should be taken into account together with the impact of age as depicted in Table 4 and Figure 2.

In the literature, the issue of sex is controversial. In a cross-sectional study of 4642 patients from the institute of antral Gujarat, it was found that the prevalence of dental caries was the highest in the male population (54.8%) [33]. However, other studies found that females had a higher percentage [37]. This study included young to middle-aged populations and a higher prevalence of SOS teeth could be attributed to more dental trauma among males in general and in particular among the military population.

However, there is evidence indicating that many caries risk factors that provide a sex bias, placing women at a higher caries risk than men [38]. These factors may include the culture-based division of labor, gender-based dietary preferences [39], different salivary composition and flow rate, hormonal fluctuations, dietary habits, social roles among their family and genetic variations [40]. Genome-wide association studies have found caries susceptible and caries protective loci, some of which are X-linked, that influence variation in taste, saliva and enamel proteins, affecting the oral environment and the microstructure of enamel, which may partly explain sex differences in caries [39]. On the other hand, a recent systematic review has shown that sex differences have narrowed over the past 20 years and were not significant in 2010. This may be related to several factors, including societal and cultural changes, improvement in female education, increased focus on women’s health and potentially even improved nutrition [41].

### 4.3. Strength and Limitations

The main strengths of the present study are the large sample size (12,146 subjects with SOS teeth and a total of 132,323 subjects comprising the study population) as well as the strict protocol utilizing dental and socio-demographic databases. Definitions were uniform for all patients. For dental parameters, both clinical examination and radiographic assessment were included. 

Limitations of this study include the cross-sectional study design, we cannot assume causality, and therefore this paper only suggests associations and correlations between the variables. 

The records included dental examinations that were conducted on all IDF military who went through a uniform training and followed a strict protocol of the dental branch of the IDF medical corps. However, optimal calibration was not conducted and there could be possible variations in the diagnosis of carious lesions and their decisions regarding treatment priorities. 

Dental examinations in the IDF are accessible and free and are mandatory for combats during their first four months of military training. However, there are cases of refusal, missed examinations or treatments in civilian dental clinics, which might cause under documentation.

Due to the complexities of the issues, and the limitation of one paper to address all issues, in the papers from the DOME database we will analyze the association of SOS teeth with multiple parameters such as lifestyle habits, medical background and dental attendance patterns. Additional studies, including long term longitudinal population-based epidemiological surveys in other settings and populations, would help address these issues.

## 5. Conclusions

Assessment of first priority SOS teeth should be part of the dentist’s work-up. Dentists and dental health authorities should design strategies to address the challenge of reducing the number of SOS teeth. The results indicate a great need for thoroughly planned prevention and restoration of dental caries and the vigilance of a higher standard of personal oral hygiene and dental check-ups are necessary to obtain an improvement of oral status in the future adult population and to reach the new WHO global goals.

## Figures and Tables

**Table 1:** Descriptive statistics of SOS teeth among the study population.

Parameter	N
Study population	132,323 (100%)
Number of patients with SOS teeth	12,146 (9.18%)
Number of SOS teeth	18,300
Number of SOS teeth per diseased patient	1.5
Mean number of SOS teeth per patient ± SD	0.14 ± 0.52
Range of SOS teeth per patient	0–20
Std. Error of Mean	0.001

**Table 2:** Distribution of the number of SOS teeth per patient among the study population.

Number of SOS Teeth per Patient	Frequency	Valid Percent	Cumulative Percent
0	120,177	90.8	90.8
1	8323	6.3	97.1
2	2438	1.8	99.0
3	829	0.6	99.6
4	328	0.2	99.8
5	141	0.1	99.9
6	50	0.0	100.0
7	18	0.0	100.0
8	14	0.0	100.0
9	2	0.0	100.0
10	1	0.0	100.0
11	1	0.0	100.0
20	1	0.0	100.0
Total	132,323	100.0	

**Table 3:** Mean number of SOS teeth and the rate of SOS teeth per 1000 by age.

Age	Number of Patients	Mean Number of SOS Teeth	Standard Deviations	Rate of SOS Teeth per 1000
18	32,130	0.16	0.575	160
19	30,330	0.15	0.568	152
20	23,254	0.13	0.504	130
21	12,295	0.13	0.493	126
22	5961	0.13	0.488	128
23	3555	0.13	0.481	132
24	2644	0.12	0.444	116
25	2150	0.13	0.514	133
26	1848	0.14	0.516	138
27	1695	0.13	0.478	132
28	1391	0.10	0.394	98
29	1184	0.14	0.463	136
30	1097	0.11	0.422	108
31	974	0.12	0.448	121
32	949	0.10	0.386	97
33	856	0.10	0.375	102
34	871	0.10	0.392	104
35	893	0.10	0.362	95
36	808	0.07	0.295	69
37	858	0.11	0.462	114
38	818	0.12	0.456	116
39	767	0.12	0.433	121
40	738	0.12	0.424	118
41	706	0.09	0.414	92
42	740	0.09	0.336	88
43	719	0.09	0.353	88
44	605	0.07	0.313	69
45	503	0.05	0.266	54
46	372	0.08	0.313	83
47	267	0.03	0.182	26
48	162	0.12	0.443	123
49	115	0.04	0.205	43
50	68	0.06	0.293	59
Total	132,323	0.14	0.522	

**Table 4:** The mean number of SOS teeth and the rate of SOS teeth per 1000 by age and sex.

Age	Female				Male			
	Number of Patients	Mean Number of SOS Teeth	Standard Deviations	Rate of SOS Teeth per 1000	Number of Patients	Mean Number of SOS Teeth	Standard Deviations	Rate of SOS Teeth per 1000
18	7812	0.13	0.489	126	24,318	0.17	0.600	172
19	10,671	0.12	0.480	123	19,659	0.17	0.609	167
20	6679	0.11	0.458	114	16,575	0.14	0.522	137
21	2145	0.10	0.400	97	10,150	0.13	0.510	132
22	1033	0.10	0.419	103	4928	0.13	0.501	133
23	618	0.09	0.379	86	2937	0.14	0.500	142
24	502	0.11	0.454	106	2142	0.12	0.441	118
25	373	0.08	0.326	80	1777	0.14	0.544	145
26	346	0.12	0.481	124	1502	0.14	0.524	141
27	336	0.10	0.348	101	1359	0.14	0.505	139
28	254	0.10	0.380	98	1137	0.10	0.397	99
29	212	0.14	0.434	142	972	0.13	0.469	135
30	211	0.09	0.312	85	886	0.11	0.444	113
31	215	0.13	0.465	130	759	0.12	0.444	119
32	181	0.15	0.525	155	768	0.08	0.344	83
33	169	0.07	0.280	71	687	0.11	0.395	109
34	170	0.09	0.312	94	701	0.11	0.409	107
35	133	0.07	0.281	68	760	0.10	0.374	100
36	135	0.05	0.331	52	673	0.07	0.287	73
37	120	0.18	0.513	175	738	0.10	0.453	104
38	120	0.12	0.434	117	698	0.12	0.460	116
39	107	0.07	0.344	65	660	0.13	0.446	130
40	70	0.13	0.479	129	668	0.12	0.419	117
41	88	0.09	0.560	91	618	0.09	0.390	92
42	85	0.02	0.152	24	655	0.10	0.352	96
43	66	0.11	0.356	106	653	0.09	0.353	86
44	54	0.07	0.264	74	551	0.07	0.317	69
45	49	0.02	0.143	20	454	0.06	0.276	57
46	30	0.07	0.254	67	342	0.08	0.318	85
47	25	0.00	0.000	0	242	0.03	0.191	29
48	16	0.06	0.250	63	146	0.13	0.459	130
49	6	0.00	0.000	0	109	0.05	0.210	46
50	7	0.00	0.000	0	61	0.07	0.309	66
Total	33,038	0.12	0.460	2886	99,285	0.15	0.542	3587

## Abbreviations

CPR	computerized patient record system
DMF	decayed, missed, filled teeth
DOME	Dental, Oral, Medical Epidemiological
DPR	Dental Patient Record
EDR	Electronic dental record
EMR	Electronic medical record
IDF	Israel Defense Forces
